# Natural Human Immunity Against Staphylococcal Protein A Relies on Effector Functions Triggered by IgG3

**DOI:** 10.3389/fimmu.2022.834711

**Published:** 2022-03-11

**Authors:** Elena Boero, Ana Rita Cruz, Werner Pansegrau, Cinzia Giovani, Suzan H. M. Rooijakkers, Kok P. M. van Kessel, Jos A. G. van Strijp, Fabio Bagnoli, Andrea G. O. Manetti

**Affiliations:** ^1^ GSK, Siena, Italy; ^2^ Department of Medical Microbiology, University Medical Center Utrecht, Utrecht University, Utrecht, Netherlands

**Keywords:** IgG3, phagocytosis, SpA, *S. aureus*, monoclonal antibodies

## Abstract

Staphylococcal protein A (SpA) is a multifunctional, highly conserved virulence factor of *Staphylococcus aureus*. By binding the Fc portion of all human IgG subclasses apart from IgG3, SpA interferes with antibody and complement deposition on the bacterial surface, impairing staphylococcal clearance by phagocytosis. Because of its anti-opsonic properties, SpA is not investigated as a surface antigen to mediate bacterial phagocytosis. Herein we investigate human sera for the presence of SpA-opsonizing antibodies. The screening revealed that sera containing IgG3 against SpA were able to correctly opsonize the target and drive Fcγ receptor-mediated interactions and phagocytosis. We demonstrated that IgG3 Fc is significantly more efficient in inducing phagocytosis of SpA-expressing *S. aureus* as compared to IgG1 Fc in an assay resembling physiological conditions. Furthermore, we show that the capacity of SpA antibodies to induce phagocytosis depends on the specific epitope recognized by the IgGs on SpA molecules. Overall, our results suggest that anti-SpA IgG3 antibodies could favor the anti-staphylococcal response in humans, paving the way towards the identification of a correlate of protection against staphylococcal infections.

## Introduction

*Staphylococcus aureus* is a Gram-positive human commensal and pathogen causing a broad variety of community and healthcare-associated infections (1). *S. aureus* diseases are becoming increasingly difficult to treat due to acquired multi-drug resistance, especially to methicillin and more recently to vancomycin (methicillin/vancomycin-resistant *S. aureus* - MRSA, and VRSA) (2, 3).

Neutrophils, the predominant phagocytic cell type of the innate response, together with monocytes and macrophages, are responsible for the initial clearance of *S. aureus* from tissues (4–6). Phagocytosis is more effective when the bacterial surface is tagged with host opsonins such as antibodies and products derived from the activation of the complement cascade, which broaden the specificity of non-self-recognition. Opsonins interact with neutrophils *via* opsonin receptors such as Fc-gamma receptors (FcγRs) and complement receptors (CRs), whose engagement initiates the phagocytic machinery. The importance of opsonins in the host defense of *S. aureus* is emphasized by studies showing that C3 deficiencies (7), neutrophil dysfunctions (8, 9), and the expression of certain FcγRs alleles (10) render patients more susceptible to staphylococcal infections, suggesting that opsonophagocytosis is a crucial mechanism for staphylococcal clearance (4, 5, 9, 11–13).

*S. aureus* evolved an arsenal of immune evasion proteins to contrast every phase of phagocytosis (5, 13, 14). Among those proteins of *S. aureus* specifically aimed at impairing the deposition of complement and antibodies (15, 16), Staphylococcal Protein A (SpA) stood out as a critical virulence factor (16–22). SpA also gained interest as a potential vaccine and immunotherapy target (23, 24), since in studies about active and passive immunization of animal models with the detoxified version of SpA, it was shown protection upon a subsequent challenge (25, 26).

SpA is a multifunctional, highly conserved 42 kDa protein (27) expressed by most *S. aureus* strains (28). SpA interacts with antibodies *via* 4-5 highly homologous Ig-binding domains on its N-terminus. Each Ig-binding domain is composed by a three α-helices bundle bearing two binding sites: the Fc-binding site, which is specific for two symmetric sites of the CH2-CH3 elbow of the Fc of human IgGs (29); and the VH3-binding site, which is specific for the framework region of B-cell receptors (29). The affinity of SpA for the Fc and VH3 of immunoglobulins is not clearly stated in the literature, but is generally considered to be in the picomolar and nanomolar range respectively (30–32). Remarkably, the Fc region of most allotypes of human IgG3 subclass does not interact with SpA, due to the presence of an Arg^435^, instead of the His^435^ found in IgG1, IgG2, IgG4, and IGHG3*18-23 allotypes (33, 34). Studies conducted mutating IgG1 H435R and IgG3 R435H confirmed the pivotal role of Arg^435^ in abrogating interaction with SpA (35). Besides immunoglobulins, SpA binds to other proteins including the Tumor Necrosis Factor α Receptor (TNFR) (36) and the *von Willebrand* factor (vWF) (37).

SpA IgG-binding properties are the most relevant in pathogenesis, driving immune suppression on multiple fronts: they impair bacterial phagocytosis (38, 39), as well as the development of anti-staphylococcal immunity (17, 40). In fact, when SpA is released as a soluble protein, it acts as a B cell immunomodulator, provoking the expansion of VH3-idiotype cells and dominating the antigen specificity of produced antibodies (17, 41). As an LPXTG cell wall-anchored protein, SpA sequesters antibodies on the bacterial surface by binding their Fc portion, thus orienting them incorrectly. Consequently, SpA prevents proper opsonization of the bacterium by impairing the Fc-Fc clustering that leads to hexamers formation and the recruitment of C1q (42) or engagement of Fcγ-Receptors (38), interrupting the classical pathway of complement deposition and FcγR-mediated phagocytosis.

Virtually all individuals come into contact with *S. aureus* during their early life and develop heterogeneous humoral responses against its antigens, including SpA (43–49). The humoral response against SpA is under-investigated, probably because of the technical challenges posed by SpA Ig-binding properties. Anti-SpA antibodies are generally detected using the mutated version of SpA [SpA_KKAA_, first described by Kim et al., 2010 (26)], impairing the Fc- and VH3-binding sites, or SpA peptides lacking the Ig-binding sites. However, the absence of Fc-sequestering sites on the mutant protein prevents us from understanding whether anti-SpA antibodies can opsonize the wild-type protein. It is necessary to distinguish *non-immune binding*, i.e. without the involvement of their antigen-binding sites, from *immune binding* i.e. binding of the antibody *via* its complementarity-determining regions (CDRs) while exposing its free Fc to interact with immune proteins and receptors (50).

In this paper, we investigate natural opsonizing antibodies targeting SpA in human serum, with a particular focus on the role of human subclasses IgG1 and IgG3 (51). In tested sera, we observed that natural antibodies against SpA wild type predominantly belong to subclass 3. Anti-SpA model mAbs expressed in IgG1 and IgG3 scaffolds tested in functional assays confirmed that IgG3 anti-SpA antibodies outperform IgG1s in inducing phagocytosis using both THP-1 monocytes and human neutrophils. Furthermore, we show that the position of binding site of specific anti-SpA IgG3 on SpA molecules affects the capacity of these antibodies to opsonize saturated bacteria. These data add important information to our knowledge of the anti-SpA immune response and suggest the role of IgG3 and its binding site position as a potentially relevant factor concurring to define a humoral correlate of protection against *S. aureus* infections, and the possible use of monoclonal antibodies as therapeutics against infectious diseases.

## Results

### Human Sera Contain Opsonizing Antibodies Against Wild-Type Staphylococcal Protein A.

To investigate the presence of opsonizing antibodies against SpA wild type (SpAwt) in human sera we used a Luminex-based assay. Briefly, beads coupled with SpAwt were incubated with human sera to allow antibody deposition. SpAwt-opsonizing antibodies would display their Fc portions, allowing their detection *via* a biotinylated soluble Fcγ-receptor IA (sFcγRIA). Finally, sFcγRIA binding would be revealed by streptavidin-coupled phycoerythrin (SAPE).

**Figure 1:**
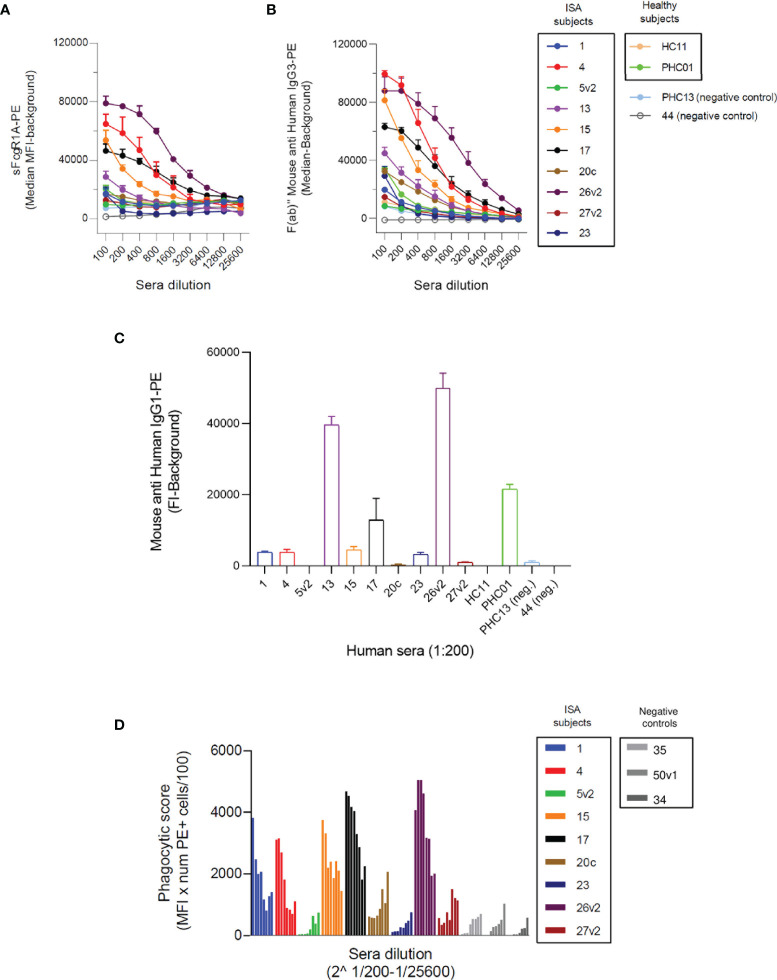
Opsonizing anti-SpA antibodies in human sera are predominantly IgG3. **(A)** Detection of SpA opsonizing antibodies in sera *via* soluble biotinylated FcγR1A coupled with streptavidin-PE. Each line represents the titration-dependent trend of median fluorescence for each tested serum. ISA subjects are identified by a progressive number, with v2 indicating a second visit. Healthy subjects are identified by the nomenclature HC = *healthy control* or PHC = *pediatric healthy control.* Two exemplificative negative controls are shown, PHC13, and 44. The data represent the mean ± SD of three independent experiments. **(B)** Detection of subclass 3 opsonizing antibodies deposited on SpAwt coated beads from positive FcγR1A hits. The data represent the mean ± SD of three independent experiments. **(C)** Detection of subclass 1 anti-SpA opsonizing antibodies deposited on SpAmut coated beads. The data represent the mean ± SD of three independent experiments. **(D)** Phagocytic score (calculates as the mean fluorescence intensity of cells of PE+ cells, multiplied by the number of PE+ cells, divided by 100) indicating the efficiency of internalization of SpAwt beads by THP-1 monocytes for a subgroup of opsonizing sera from ISA patients used, compared to three representative anti-SpA IgG negative sera 35, 50v1, 34 in gray. The data represent one experiment.

Sera and plasma samples from 116 *S. aureus*-infected subjects or healthy controls were screened for the presence of anti-SpAwt opsonizing antibodies (**Table 1**). Only 12 samples (10.34%) displayed anti-SpAwt opsonizing antibodies. Invasive *S. aureus* (ISA) disease patients displayed the highest frequency of positive sera (23.25%) and the highest sFcγRIA signals. In contrast, only 2 hits were identified in the healthy control (HC) group (4.17%) (**Table 1**). If we take into account the ISA patients only, the presence of IgG anti-SpA was more associated with this group (OR=6.97, CI 95% CI 1.43, 33.93, p<0.016). **Figure 1A** shows the concentration-dependent profile of the median fluorescence of the sFcγRIA signal for all 12 positive sera. Positive sera were defined by relative positivity and their titration profile, as described in materials and methods and in supplementary (**Figure S1**).

**Table 1:** Details of anti-SpA antibody screening in patients.

Category	Total individuals (number)	SpA-positive individuals (number)	SpA-positive individuals (percentage)
*ISA*	43	10	23.25%
*Others infection types*	25	0	0.00%
*HC*	48	2	4.17%
*Total*	116	12	11.21%

The table describes the *S. aureus* infection status of screened individuals; invasive S. aureus disease patients (ISA) and other infection types or healthy controls (HC). Number of individuals for each group is indicated, and serum positivity for anti-SpA opsonizing antibodies in terms of both number of individuals within groups and percentages of groups are also shown.

Since Fcγ-receptor IA recognizes IgGs of subclass 1, 3, and 4, and knowing that IgG4 are particularly involved in the immune response to allergens (52, 53), we wondered whether anti-SpA opsonizing antibodies belonged to subclass IgG1 and/or IgG3. First, we evaluated the presence of IgG3 on serum-opsonized SpAwt beads. IgG3 screening revealed a high content of IgG3 (**Figure 1B**), especially in the sera with high sFcγRIA signal. Since IgG3 is the second least abundant IgG subclass in serum, we checked whether these individuals overproduced IgG3, but they all displayed average levels of IgG3 (**Figure S2**). Afterward, since identification of IgG1 opsonizing antibodies on SpAwt does not provide clear results, probably due to the non-immune binding of IgG1 anti-SpA antibodies to SpA (**Figure S3**), we screened for IgG1 antibodies with the mutant version of SpA (SpAmut). Indeed, we also observed the presence of anti-SpA IgG1 in positive sera, especially in 26v2, 13, PHC01, and 17 (**Figure 1C**).

To confirm the interaction between the free Fc portions of opsonizing anti-SpAwt antibodies with FcγR1 in a physiological model, we set up a phagocytosis assay with THP-1 cells, a human monocytic cell line commonly used as a model of FcγR-mediated phagocytosis. **Figure 1D** shows the efficiency of SpAwt beads phagocytosis induced by a subgroup of positive sera. The sera with the highest sFcγR1 and αIgG3 signals in **Figures 1A, B** mediated the internalization of beads. Sera with lower or borderline sFcγR1 and αIgG3 signal on the contrary did not induce phagocytosis, suggesting that opsonizing IgG3 might be important to support the internalization of SpAwt targets by phagocytes.

In summary, we showed that anti-SpAwt opsonizing antibodies with available Fc for effector function are present in a small percentage of subjects, especially those who faced an invasive disease. These sera contain IgGs predominantly belonging to subclass IgG3. Some positive sera also display IgG1 anti-SpAmut titers, but it is unclear whether these antibodies concur in inducing Fc-mediated effector functions. Sera with relatively high anti-SpA IgG3 titers are capable of mediating phagocytosis of SpAwt beads in a THP-1 phagocytosis model. Sera containing low levels of IgG3 do not exert the same phagocytic stimulus, encouraging a more extensive investigation of the impact of IgG3 versus IgG1 subclass on the capacity of SpA specific antibodies to opsonize their target.

### Anti-SpA IgG3 Mediate Successful FcγR-Mediated Internalization of SpAwt Beads and *S. aureus* by THP-1

In the previous section we showed that SpA-specific IgG3 were able to engage FcγRs in the presence of SpA, while it was not clear whether IgG1 contributed to the induction of Fc-mediated effector functions. To better investigate the functionality of IgG3 versus IgG1 anti-SpA antibodies, we expressed four publicly available anti-SpA monoclonal antibodies (mAbs) in IgG1 and IgG3 scaffolds (21, 54) (**Figure S10**).

**Figure 2:**
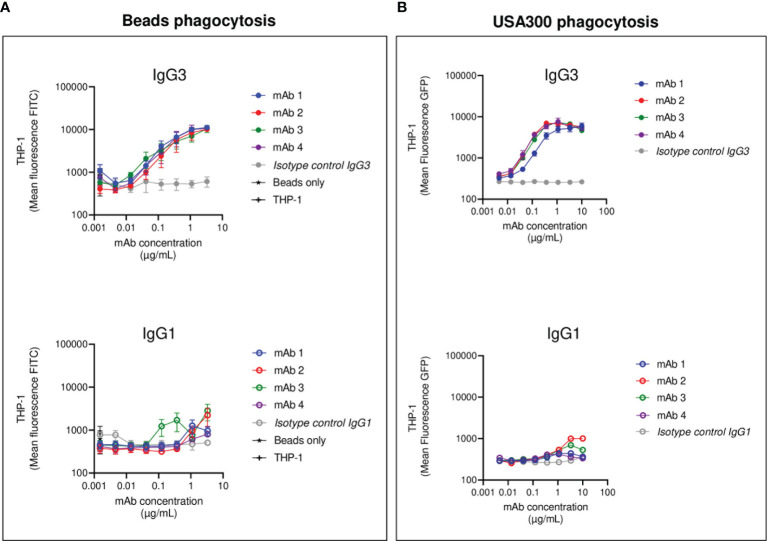
THP-1 phagocytosis of SpAwt beads and USA300 lac opsonized with anti-SpA IgG3 and IgG1 mAbs. Mean fluorescence of total THP-1 population engulfing fluorescent FITC-labelled SpAwt beads **(A)** or GFP-expressing *S. aureus* USA300 lac **(B)** opsonized with increasing concentrations of four model anti-SpA antibodies. In the upper panels, targets are opsonized with mAbs expressed in the IgG3 scaffold (*full dots*), while the lower panels targets are opsonized with mAbs expressed in the IgG1 scaffold (*empty dots*). The data represent the mean ± SEM of three independent experiments.

Each mAb with IgG1 or IgG3 scaffold was used to opsonize SpAwt on beads and bacteria in a THP-1 model of FcγR-mediated phagocytosis. Successful phagocytosis would indicate that the Fc portion of the opsonizing mAb can opsonize SpA as well as engage Fc-receptors, despite the presence of SpA Ig-binding domains. All four IgG3 anti-SpA mAbs induced efficient phagocytosis of fluorescent targets in a dose-dependent manner, as indicated by the mean fluorescence (MFL) of the phagocytosing cells reaching a plateau for both beads (**Figure 2A**) and bacteria (**Figure 2B**). Virtually all THP-1 participated in the uptake (**Figures S4**, **S5**). In contrast, when beads or bacteria were opsonized with the IgG1 version of each mAb, uptake was less efficient (**Figures 2A, B** and **Figures S4**, **S5**), despite the deposition of IgG1 on bacteria (**Figure S6**). Opsonization with high concentrations of IgG1 mAb-1 and mAb-2 induced limited internalization and in less than 50% of THP-1 cells (**Figures S4**, **S5**).

In summary, anti-SpA IgG3 mAbs mediate efficient internalization of targets, since their Fc is free to interact with cell receptors despite the presence of SpA. On the other hand, IgG1 drive limited phagocytosis of targets by THP-1 cells, indicating that the availability of their Fc is impaired by the presence of SpAwt. However, some IgG1 retain the ability to engage FcγRs to a low extent.

### IgG3 Opsonize SpAwt and Activate Complement in a Human Neutrophil Phagocytosis Model

After having investigated the ability of IgG1/IgG3 anti-SpAwt mAbs to induce FcγRs-mediated phagocytosis in THP-1, we assessed the ability of anti-SpAwt antibodies to induce FcγRs- and complement-mediated phagocytosis in human primary neutrophils. The use of neutrophils and complement can provide us with additional information about the engagement of Fcs. Neutrophils present a different array of phagocytic FcγRs compared to THP-1 (55) and express phagocytic complement receptors CR1 and CR3, whose engagement would indicate the successful deposition by the Fc-dependent classical pathway of complement deposition (56, 57).

**Figure 3:**
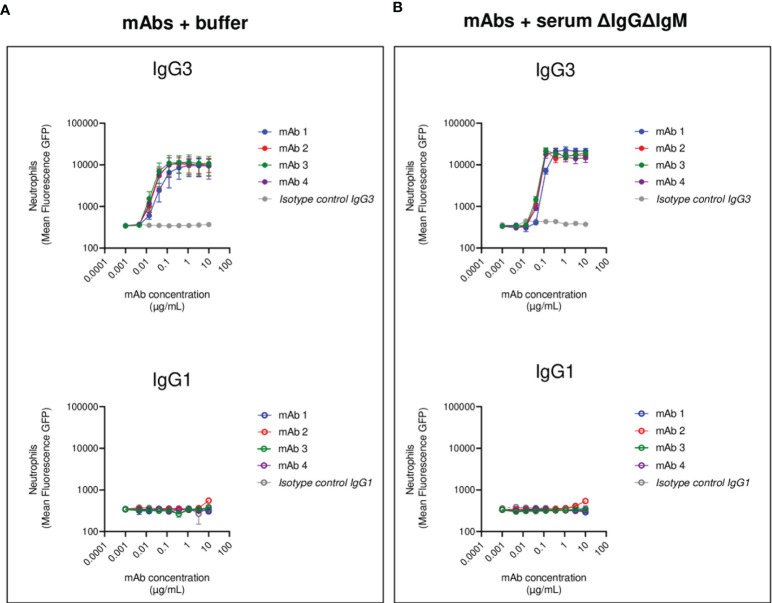
Human neutrophils phagocytosis of USA300 opsonized with anti-SpA IgG3 and IgG1 mAbs and human complement. Mean fluorescence of total neutrophil population engulfing *S. aureus* USA300 opsonized with anti-SpA mAbs in the absence **(A)** or presence **(B)** of a human complement source depleted of IgG and IgM (ΔIgGΔIgM serum). In the upper panels, bacteria are opsonized with mAbs expressed in the IgG3 scaffold (*full dots*), while in the lower panels, bacteria are opsonized with mAbs expressed in the IgG1 scaffold (*empty dots*). The data represent the mean ± SEM of three independent experiments.

First, we measured FcγRs-mediated phagocytosis. Fluorescent *S. aureus* was opsonized with each mAb pair and incubated with human neutrophils. All four anti-SpA mAbs in IgG3 form induced strong phagocytosis with a lower effectivity for mAb-1, confirming the observations made in the THP-1 phagocytosis model (**Figure 3A**). Only IgG1 mAb-2 showed a minimum stimulation of uptake compared to negative controls, and in less than 20% of the cells (**Figure S7**).

We further investigated Fc-Fc interactions in the context of complement-mediated phagocytosis. The first step in the activation of the classical pathway of complement is the multimerization of antibodies at the Fc-Fc interface, followed by the engagement of the C1 complex (58). Both events indirectly indicate that interaction among Fc regions happens. Our group already demonstrated that SpA prevents the successful hexamerization of antibodies by binding antibodies in the Fc-Fc interface, thus disrupting complement deposition (42). *S. aureus* was opsonized with each mAb in the presence of a human complement source made from serum depleted of complement-fixating antibodies (denoted as ΔIgGΔIgM serum) (59). The presence of complement enhanced IgG3-mediated phagocytosis, as suggested by the steep increase in the MFL of neutrophils (**Figure 3B**). In contrast, the presence of complement did not improve the internalization of IgG1 opsonized bacteria (**Figure 3B**).

In summary, the results obtained in the neutrophil phagocytosis model confirmed and reinforced the results observed with THP-1 cells. Anti-SpAwt IgG3 mAbs were always able to induce phagocytosis, engaging FcγRs and organizing in multimers that interacted with the C1 complex. On the contrary, IgG1 anti-SpAwt did not stimulate uptake, suggesting that IgG Fcs were neither interacting with FcγRs on neutrophils nor organizing in multimers that allowed successful complement deposition because of the presence of SpA.

### Anti-SpA IgG3 mAb Induce Phagocytosis, Even in the Presence of Non-Specific Abs Bound to SpA

In the previous sections, we investigated the ability of anti-SpAwt mAbs to induce phagocytosis of *S. aureus*. However, in physiological conditions, *S. aureus* is exposed to human IgGs that are sequestered by SpA Ig-binding sites. Therefore, we next tested the ability of anti-SpA mAbs to induce phagocytosis in the presence of normal human IgGs.

**Figure 4:**
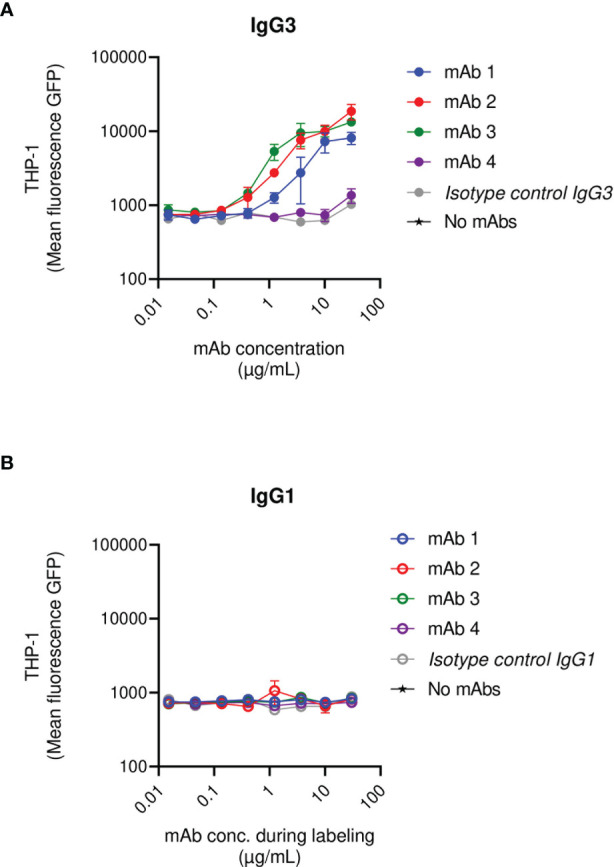
THP-1 phagocytosis of USA300 lac opsonized with anti-SpA IgG3 and IgG1 mAbs in the presence of human IgGs. Mean fluorescence of total THP-1 population engulfing *S. aureus* USA300 opsonized with anti-SpA mAbs in IgG3 scaffold **(A)** or IgG1 scaffold **(B)** in the presence of a saturating concentration of human IgGs. The data represent the mean ± SEM of three independent experiments.

Briefly, fluorescent *S. aureus* was pre-exposed to a saturating concentration of human IgGs and was subsequently opsonized with a titration of the anti-SpAwt IgG3 or IgG1 mAbs. When saturated bacteria were incubated with the IgG3 isotype control they were not phagocytosed, demonstrating that, at the concentration of human IgGs used, natural anti-staphylococcal antibodies did not induce phagocytosis *per se*. Anti-SpA IgG3 mAbs 1-3 still displayed the ability to induce uptake by THP-1. Interestingly IgG3 mAb-4 completely lost its ability to induce phagocytosis in the presence of interfering human antibodies (**Figure 4A** and **Figure S8**). In line with previous results, IgG1 mAbs had limited ability to induce phagocytosis in the presence of interfering human IgGs (**Figure 4B**).

In summary, most anti-SpAwt IgG3s correctly opsonize SpA, even when saturated by human IgGs. The presence of non-specific antibodies bound to SpA rendered the opsonization of SpA more stringent. In these conditions, the binding site of the mAbs appears to play a more prominent role.

### Only Some Anti-SpA IgG3 mAbs Displace Fc-Sequestering Human IgGs

**Figure 5:**
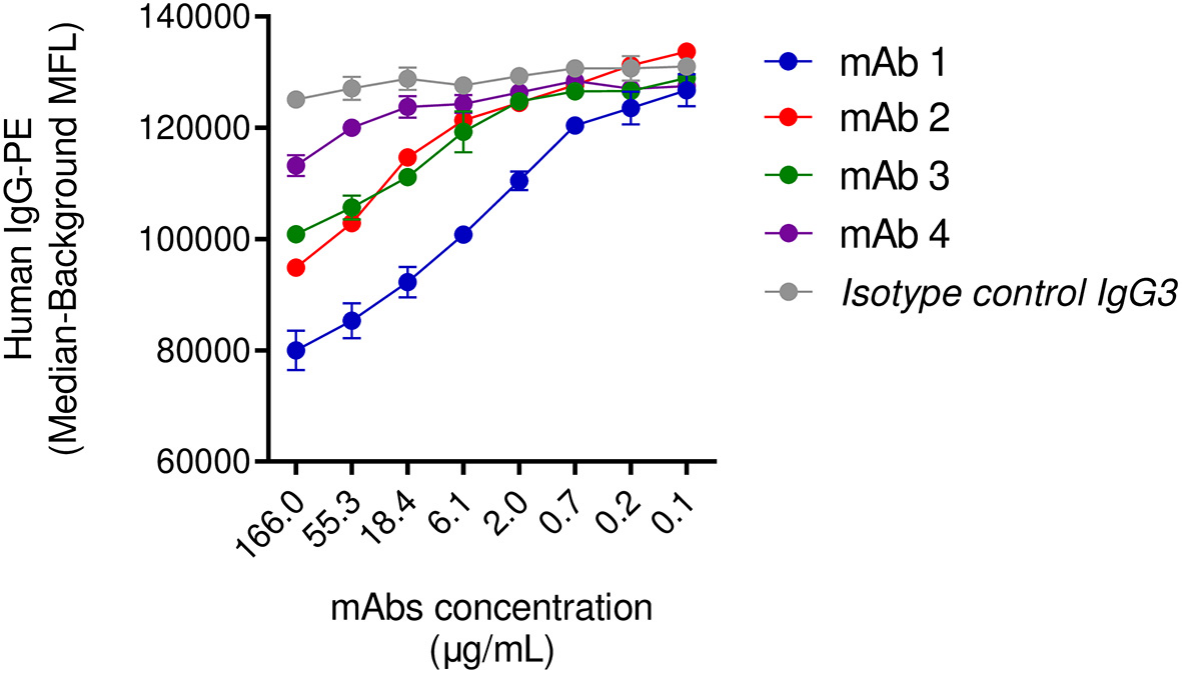
Displacement of human IgGs from SpAwt beads by anti-SpA monoclonal antibodies. Mean fluorescence associated to PE-conjugated human IgGs attached to SpA wild type on beads after incubation with IgG3 anti-SpA monoclonal antibodies and an isotype control (**Figure S10**). The data represent the mean ± SEM of two independent experiments.

In the previous sections, we highlighted the inability of IgG3 mAb-4 to induce phagocytosis in the presence of human IgGs. To corroborate our observation we investigated the dynamic of the binding of SpAwt opsonizing antibodies to SpA when it is saturated with human antibodies. We set up a displacement assay in which Luminex SpAwt-beads were saturated with fluorescently-labeled human IgGs (Hu-IgGs) and subsequently incubated with increasing concentrations of each mAb. A decrease in the fluorescence associated with the Hu-IgGs-PE on the beads would indicate that mAbs can detach human IgGs from SpA. **Figure 5** shows that all mAbs tested apart from mAb-4 reduced the number of human IgGs attached to SpAwt beads compared to the isotype control. mAb-1 shows an enhanced capacity of displacing human antibodies from SpA as compared to mAb-2 and mAb-3.

It is possible that mAb-4 fails in inducing phagocytosis in the presence of human IgGs and in displacing IgGs because of its epitope recognition on SpA, which might be in an unfavorable position. Since the model mAb epitopes are not known, we performed an approximate mapping based on the interference by the SpA_AA_ and SpA_KK_ mutations, located in the SpA VH3 and main Fc binding sites, respectively (26). If the CDR interaction with a given mAb, using the IgG3 scaffold, was abolished by the presence of one of the mutations, we concluded that the epitope would overlap with that mutation site or be affected by a conformational change due to the mutation. If no interference was observed, no information about the epitope location could be gathered (**Figure S9**).

As reported in **Table 2**, mAb-1 only binds to the SpA_KK_, indicating that its epitope is destroyed by the mutations in the VH3-binding region. mAb-2 and mAb-4 only bind to the SpA_AA_ mutants, indicating that their epitopes overlap with the Fc-binding region of SpA. Finally, mAb-3 binds both mutants, suggesting that its epitope lays outside both antibody binding regions of SpA or the mutations do not interfere with mAb binding, leaving the epitope intact. These results suggest that epitope location could be relevant for the ability to induce opsonophagocytosis of the bacterium.

**Table 2:** Approximate epitope mapping of IgG3 anti-SpA mAbs using SpA_AA_ and SpA_KK_ mutant proteins.

mAb	Binds to:	Approximate epitope location
**mAb-1**	SpA_KK_	SpA VH3-binding region
**mAb-2**	SpA_AA_	SpA Fc-binding region
**mAb-3**	SpA_AA_ and SpA_KK_	unknown
**mAb-4**	SpA_AA_	SpA Fc-binding region

In summary, this experiment shows that anti-SpAwt IgG3 mAb-4 cannot outcompete sequestered human IgGs, probably explaining impaired SpA opsonization and the inability to drive phagocytosis in the previous section. The mapping experiments were inconclusive with respect to the reason for the impairment of mAb-4.

## Discussion

Correct opsonization of *S. aureus* by antibodies is a pivotal step of staphylococcal clearance (4, 5, 9). Several cell wall-anchored proteins are described as potential targets for opsonizing antibodies (60), but this is controversial for SpA. Offering additional high-affinity binding sites for the Fc region on the bacterial surface, SpA severely interferes with antibody deposition, ultimately hampering opsonophagocytosis (42).

The group of Olaf Schneewind extensively demonstrated that SpA is a valuable staphylococcal target to be addressed by the humoral response. In fact, humoral immunity against SpA, achieved either by active immunization with the detoxified version of SpA (SpA_KKAA_) or by administering anti-SpA monoclonal antibody 3F6 provided protection in a mouse model of blood infection (20, 26), and achieved mice decolonization (61). Reasonably, SpA-mediated opsonophagocytosis contributes to protection. However, it is not clear whether these results are translatable to humans. In fact, mouse IgG1, the most abundant IgG subclass in mice, is poorly sequestered by SpA. Furthermore, the SpA-sensitive mouse IgG subclasses are bound with lower avidity compared to human subclasses. Overall, mouse antibodies are sequestered by SpA less efficiently than human ones. Consequently, mouse anti-SpA IgGs are probably better at opsonizing SpA and inducing effector functions in immune cells. This may represent a critical advantage skewing the outcome in mouse models.

In this work, we investigate the anti-SpA response, with a focus on whether bearing a SpA-specific variable region provides advantages to human IgGs in the opsonization of wild-type SpA. We do so by investigating the availability of the antibodies Fc portion, which indicates the ability to induce humoral and cellular effector functions (62).

First, we screened for anti-SpA opsonizing antibodies in human sera. We found that the samples containing the opsonizing antibodies contained high titers of anti-SpA IgG3. These antibodies could engage Fc effector molecules suggesting a possible important physiological role of this low-frequency subclass in the anti-SpA response. However, due to the original design of the study, it was impossible to draw any conclusion on whether the presence of IgG3 anti-SpA antibodies in the serum of patients would improve the outcome of the disease. To our knowledge, this is the first paper highlighting the presence of anti-SpA IgG3 antibodies in human sera. The high efficiency of sera containing high titers of IgG3 in interacting with cell receptors and inducing phagocytosis is perhaps not surprising. First, IgG3 antibodies are not bound by SpA because of their Arg^435^ that abrogates Fc sequestering. Interestingly, six IgG3 allotypes frequent in Orientals and other ethnicities (33) naturally bear a His^435^ and therefore can be sequestered by SpA (35). The epidemiology and pathogenesis of *S. aureus* infections in populations with a high prevalence of homozygous individuals for these alleles would be worth investigating. Second, IgG3 antibodies display the best effector functions among the IgG subclasses thanks to their unique structural properties (63).

Our data confirm the observation that IgG3s induce the best effector functions in comparison to IgG1s, binding FcγRs with high efficiency and fixating complement even with low-density antigens. We cannot exclude that the lectin pathway of complement activation also contributes to complement deposition, however our experimental settings with 1% serum strongly favour the classical pathway-mediated deposition. In agreement with a possible role of IgG3 in the phagocytosis of SpA-expressing *S. aureus*, Whitehouse et al. showed that high titers of anti-*S. aureus* IgG3, but not IgG1 in serum of patients, correlated with binding to neutrophils (44). In a recently published work, our group used recombinant anti-WTA monoclonal antibodies to demonstrate that soluble SpA blocks hexamer formation on target surfaces. IgG3 may thus be the only subclass efficiently forming hexamers on target surfaces and driving *S. aureus* killing *via* complement deposition (22), as confirmed by our data of complement-mediated phagocytosis.

Only 1 in about 10 individuals in our cohort produced anti-SpA opsonizing antibodies, especially of subclass IgG3. Interestingly, of these, 1 out of 7.5 individuals were patients diagnosed with an invasive *S. aureus* disease, suggesting that the pathogenesis of ISA might lead to the production of anti-SpA IgG3 antibodies, as shown by the OR. The anti-SpA response varied among the sera of the same patient collected at different visits (data not shown). One possible explanation for this could be that IgG3 class-switching happens early in the infection and is often transient, as observed in previous immunological studies (34, 53). Follow-up studies could elucidate the evolution of subclass specificity of the anti-SpA response over time.

Together with specific IgG3, we also demonstrated the presence of anti-SpA IgG1s in sera. However, we could not fully elucidate whether their Fc portion was participating in the engagement of sFcγR1 in our Luminex-based assays. Further experimental efforts will be required to develop discriminative assays to elucidate the Fc-availability/orientation of IgG1 antibodies against SpA (64). To overcome this limitation and explain our observations in sera, we produced four model SpA-specific monoclonal antibodies in IgG1 and IgG3 scaffold to be tested singularly in functional assays. If they were able to induce phagocytosis of SpA-targets, this would indicate that their Fc is free to engage cell receptors, indirectly providing information over its availability and orientation.

Our results show that SpA-specific IgG1 mAbs induced limited internalization in our phagocytosis assays. Our data thus suggest that IgG1 antibodies, despite bearing a specific antigen-binding fragment, promote low effector functions in the presence of SpA. Based on these results, we could speculate over the orientation of the antibody. IgG1s might be largely sequestered *via* their Fc, with the high avidity of SpA for the Fc of IgG1s outcompeting the avidity of the CDRs for their epitope. However, it is worth noting that all four model mAbs were specifically chosen to have a comparable affinity for their epitope as SpA has for Fc regions. Another hypothesis is that anti-SpA IgG1s may be bound by both Fc and CDRs, forming a “bridge-like” structure on the bacterial surface, referred to as antibody bipolar bridging. This hypothesis might explain why we observe internalization when high concentrations of some IgG1s are used as opsonizing agents. If mAb CDRs were neutralizing SpA they would progressively occupy SpA Ig-binding domains and allow an increasingly higher deposition of opsonizing antibodies. Finally, IgG1 anti-SpA could present in mixed orientations, with available Fcs possibly limited by e.g. steric hindrance of misplaced antibodies.

The analysis of the opsonizing and neutralizing properties of our four model monoclonal antibodies also provided hints about the importance of the epitope addressed by the CDRs. In fact, not only IgG1 mAbs, but also IgG3 mAb-4 failed to opsonize SpA when pre-saturated by human IgGs. This could be due to the fact that sequestered aspecific human antibodies could shield the target epitope of the anti-SpA antibody on the Fc-binding domain. This observation demonstrates that epitope recognition is important, both for the mAbs and probably also for the IgG3 in serum. On the contrary mAb-1, the only monoclonal antibody binding to the VH3 region, shows an enhanced ability to displace human antibodies on beads. This may be partly due to the additional displacement of highly prevalent human antibodies bound in a non-immune manner *via* their framework region of VH3 type. Further studies are required to identify the best epitopes to opsonize SpA when bound by human IgGs.

In conclusion, our study uses anti-SpA mAbs to elucidate the properties of an effective anti-SpA response observed in normal human serum. Our data suggest that SpA can be a valuable antigen to target to mediate *S. aureus* phagocytosis and highlight the importance of IgG3 antibodies in overcoming SpA Fc-sequestering (42, 44). In all, these data could be important to identify a correlate of protection against *S. aureus* and to stimulate the research on new therapies against *S. aureus*-related infectious diseases. Further studies are needed to investigate a possible link between the frequency of anti-SpA or anti-*S. aureus* IgG3s and the outcome of a given *S. aureus* disease.

## Methods

### Human Sera

Study participants were prospectively enrolled at Vanderbilt University Medical Center in Nashville, TN, USA. Serum or plasma samples were obtained from patients after culture-confirmed invasive *S. aureus* disease and from healthy, uninfected subjects with no known history of *S. aureus* infection. The VUMC Human Subjects Protection Program (IRB) approved the protocol before the initiation of any clinical study procedures. The following subjects were enrolled in the study: 43 patients with invasive *S. aureus* disease (ISA: 18 with bacteremia/sepsis (3 of which were catheter-associated), 13 with musculoskeletal infection (3 of which involved prosthetic joints), 10 with endocarditis, and 2 with pneumonia) and 48 healthy subjects of which 34 were adults (indicated as healthy control, HC) and 14 were children (indicated as pediatric healthy controls, PHC).

Aliquots of serum and plasma samples were prepared at VUMC were shipped to GSK Vaccines Siena for laboratory analyses. Subsequently, aliquots of sera or plasma samples were filter-sterilized by Spin-X Centrifuge Tube Filter 0.22 µm (Costar) and centrifuged for 1 min at 12,000 xg at room temperature. Filtered samples were heat-inactivated at 56°C for 30 minutes to denature key proteins of the complement cascade, aliquoted, and stored at -20°C until use.

### Expression and Purification of Anti-SpA Monoclonal Antibodies

The DNA strings encoding the variable region (V) of the heavy (H) and light (L) chains of human monoclonal antibodies, codon-optimized for mammalian expression, were synthesized by Geneart (Life Technologies) The aminoacidic sequences of each antibody are attached as supplementary material (**Figure S10**).

The recombinant mAbs were expressed in mammalian cells Expi293 (Thermo Fisher) by transient transfection in suspension, powered by the cationic lipid-based ExpiFectamine 293 transfection reagent in combination with specialized transfection enhancers. Equal amounts (15 μg each/30 mL of transfection volume) of IgH and corresponding IgL chain expression vector DNA were used. Cells were centrifuged at 350 xg for 10 minutes after 3 and 6 days after transfection. Afterward, the supernatant was collected and filtered using a 0.22 μm filter (Millipore) to remove cell debris.

Recombinant full IgGs were purified by protein G affinity chromatography using a HiTrap Protein G HP (GE Healthcare) on ÄKTA Purifier (ÄKTA). mAbs were eluted with 0.1 M glycine (pH 2.7) in tubes containing 1/10 v/v 1 M Tris-HCl (pH 9.0) as a neutralizer. After elution, the antibodies were immediately exchanged into PBS buffer using Zeba Spin desalting columns (Thermo Scientific) and were quantified by absorbance at 280 nm by NanoDrop spectrophotometer (ThermoFisher). Protein purity was assessed by SDS-PAGE after Coomassie staining (Problue Safe Stain GiottoBiotech) in reducing and non-reducing conditions and by analytical size-exclusion chromatography performed on a Superdex Increase 200 5/150 GL (Cytiva).

### Expression and Purification of Recombinant SpA

In this paper we used four forms of recombinant SpA. All proteins were expressed and purified as previously described in (65). For more details see **Table 3**.

**Table 3:** Description of SpA recombinant proteins used in this work.

Protein	Notes	References
SpA wild type (SpAwt)	SpAwt expressed by *S. aureus* strain NCTC 8325 (**Figure S11**)	GenInfo Identifier assigned by NCBI: 88193885
SpA mutant (SpAmut)	5 domains protein mutated in both VH3- and Fc-binding region (**Figure S11**)	(66)
SpA_AA_	5 domains protein mutated in VH3-binding region	(26)
SpA_KK_	5 domains protein mutated in Fc-binding region	(26)

### Coupling of Beads With SpAwt and SpAmut

20 μg of recombinant SpA wild type or SpAmut were chemically coupled to 1.25x10^6^ MagPlex fluorescent beads (Luminex corporation). To optimize and standardize the coupling reaction an automated coupling method was used optimized for an automated liquid handling workstation (Hamilton – Microlab STAR IVD). Briefly, antigens were coupled by a two-step carbodiimide procedure during which microsphere carboxyl groups are first activated with 1-Ethyl-3-(3-dimethylaminopropyl) carbodiimide hydrochloride (EDC, Pierce), in the presence of Sulfo-NHS (Pierce) to form a sulfo-NHS-ester intermediate. The reactive intermediate is then replaced by a reaction with the primary amine of the target molecule to form a covalent amide bond.

### Luminex Screening and Subclass Detection Assay

The screening of sera was performed by an automatized procedure at the epiMotion 5057 (protocol included). Briefly, 50 µl of 2-fold serially diluted sera were incubated with 20 µl SpAwt- or SpAmut -coupled beads at a concentration of 1.25x10^6^ beads/mL for 30 min at room temperature with vigorous shaking. After washing, opsonizing antibodies were detected *via* soluble FCGR1A (Sino Biological) at a final concentration of 0.17 µg/mL. IgG3 antibodies were detected with a Monoclonal Mouse Anti-Human IgG3 Secondary Antibody (Fab’2) (LSBio) at a final concentration of 0.8 µg/mL, and the presence of IgG1 antibodies was detected with a Rat anti-Human IgG1 Heavy Constant antibody (Biolegend), at a final concentration of 0.15 µg/mL. All secondary detectors were biotinylated by reacting them with biotin-NHS (Sigma). Detection of IgG1 on SpAmut was performed with Mouse Anti-Human IgG1 Hinge-PE at a final concentration of 2 µg/mL (Southern Biotech). After washing the excess detectors, the presence of antibodies was revealed with 50 µl of a final concentration of 0.1 mg/mL PE-conjugated streptavidin (Invitrogen), and the signal was acquired with FLEXMAP 3D (Luminex Corporation). Median fluorescence intensity (MFI) was subtracted from the background signal (SpA beads + secondary antibody + streptavidin-PE) and was evaluated by using Graphpad Prism 8.1.2 (GraphPad Software Inc, CA).

The positivity of sera was judged by estimating whether successive fluorescence points respected the expected trend of dilution steps. A ratio between two consecutive fluorescence points-background is calculated, to reflect the linearity assumption expected from sample 2fold dilution. At least two consecutive concentrations with MFI Ratios within 0.55 – 1.4 define a positive serum. Fluorescent points for each serum were fitted in a 5 parameters logistic curve and R^2^ was shown. Data were analyzed with Excel Version 2008 and JMP version 14.2.

### Bacterial Strains Used and Growth Procedure

USA300 lac expressing GFP was kindly gifted by Tim Foster, Trinity College. Glycerol stocks were plated overnight on Tryptic soy agar plates at 37°C. Single colonies were then grown overnight in 3 mL Tryptic soy broth (TSB) to synchronize growth and SpA expression. Finally, overnight growths were restarted in 30 mL TSB from A_600_ 0.05 to mid-exponential phase A_600_ 2. The expression of SpAwt was verified (data not shown). Bacteria were then washed in assay buffer (RPMI-BSA 0.5%, counted *via* FACS LSRII (Becton Dickinson) with FACSDiva 8.0.1 acquisition software, and resuspended to the working concentration of 3.75x10^8^ bacteria/mL. 1 mL aliquots of live bacteria were stored at -20°C upon use.

### THP-1 Cells

THP-1 (ATCC TIB-202) were cultured in RPMI 1640 medium supplemented with GlutaMax and HEPES (Gibco), 10% FBS, and 5 µg/mL penicillin/streptomycin, according to manufacturer indications. Before use, THP-1 morphology was checked and viability was measured to be above 90% *via* Vi-CELL XR Cell Viability Analyzer (Beckman Coulter). Finally, cell concentration was adjusted in assay buffer without antibiotics and FBS.

### THP-1 Phagocytosis Assays

#### Phagocytosis Assay of Serum-Opsonized Beads

10^7^ FluoSpheres NeutrAvidin-Labeled Microspheres, 1.0 µm in diameter, red fluorescent (580/605) (ThermoFisher), were saturated overnight at 4°C with 10 µg of biotinylated recombinant SpAwt (produced *in house*) in a final volume of 100 µl. After washing, 10^6^ beads were incubated with two-fold titrated sera from 1/200 for 30 minutes at 37°C with mild shaking (250 rpm). THP-1 with viability higher than 95% were then added to each well in a final ratio of 10:1 and the incubation was continued for 30 minutes. The plate was centrifuged at 300xg and cells were resuspended in a quenching solution of 0.2% trypan blue to exclude the fluorescence of adherent beads. Data from 10.000 THP-1 were acquired by flow cytometry on a BD FACS Canto II (Becton Dickinson) equipped with FACSDiva 8.0.1 and analyzed with FlowJo (Becton Dickinson, version 10.0.7 for Mac). A phagocytic score was calculated by determining the number of PE-positive cells multiplied by their mean fluorescence intensity (number PE beads-positive cells x MFI).

#### Phagocytosis Assay of mAb-Opsonized Beads

2x10^8^ Dynabeads M-280 magnetic beads, 2.8 µm in diameter, (ThermoFisher) were incubated in 2 mL 0.5 mg/ml FITC solution on ice protected from light for 30 minutes. After thorough washing in PBS, FITC Dynabeads were labeled with 25 µg/mL biotinylated SpA wild type for 30 minutes at 4°C with vigorous shaking in phagocytosis buffer, RPMI + BSA 0.05%. In a 96 well plate, 20 µl of 1x10^8^ FITC-SpA wild type beads/mL were pre-incubated with 20 µl three-fold titrated mAbs 1-4 for 30 minutes at 37°C with vigorous shaking. Then beads were diluted to the working concentration of 1.875x10^6^ beads/mL, distributed 40 µl per plate and >95% vital THP-1 were added at a final ratio of 10:1. Beads were phagocytosed for 30 minutes at 37°C with vigorous shaking. Finally, each well was added with a quenching solution of 0.2% trypan blue to exclude the fluorescence of adherent beads and read immediately at the CANTO II (Becton Dickinson) as described above.

#### Phagocytosis Assay of mAbs Opsonized USA300

20 µl of 3.75x10^7^/mL GFP-expressing bacteria were pre-opsonized in assay buffer with 20 µl of a titration of sera or mAbs, incubating for 30 minutes at 37°C with vigorous shaking. 10 µl of 7.5x10^6^ cells/mL THP-1 were added immediately, and incubation was continued for another 30 minutes. Lysostaphin was added to reach a final concentration of 10 µg/mL and incubated for a further 10 min to lyse all non-internalized bacteria. Phagocytosis was stopped on ice and samples were fixed by adding 100 µl of 2% formaldehyde and incubating on ice with shaking for 1h. Samples were acquired at FACS CANTO II (Becton Dickinson) with FACSDiva 8.0.1 software.

### Surface Plasmon Resonance

Surface plasmon resonance experiments were performed in a Biacore T200 instrument (GE Healthcare). In brief, SpA mutants SpA_AA_ and SpA_KK_ were immobilized on the surface of a CM-5 sensorchip by amine coupling using the Amine Immobilization Kit (GE Healthcare) according to the manufacturer’s instructions. To assess epitope interference of AA and KK mutations, mAbs were injected for 60 s at 100 nM and 30 µL/min in HBS-EP+ (0.01 M HEPES pH 7.4, 0.15 M NaCl, 3 mM EDTA, 0.005% v/v Surfactant P20) running buffer (GE Healthcare). Dissociation was monitored for 200 s after which the chip was regenerated by 60 s-injections of 10 mM glycine-HCl, pH 1.7. Sensorgrams were analyzed using the Biacore evaluation software version 3.0 (GE Healthcare).

### Isolation of Neutrophils From Fresh Blood

The protocol for neutrophil isolation was extensively described in (6). Briefly, fresh heparinized blood from healthy donors was diluted 1:1 in PBS and layered on a Ficoll- (GE Healthcare) Histopaque (density 1.119; Sigma) gradient to separate the polymorphonuclear cells (PMNs) from plasma and other cellular components. Residual erythrocytes were lysed by hypotonic shock and finally, PMNs were kept in RPMI-HSA (0.05%) buffer at 4°C upon use. The most abundant fraction of PMNs is represented by neutrophils (>95%) (67).

Informed consent for blood donation was obtained from all subjects, in accordance with the Declaration of Helsinki. Approval from the Medical Ethics Committee of the University Medical Center Utrecht was obtained (METC protocol 07-125/C approved on March 1, 2010).

### Neutrophils Phagocytosis Assay

The protocol for the phagocytosis assay was extensively described in (62). Briefly, 20 µl of USA300 GFP-expressing bacteria were pre-incubated with 20 µl of a titration of mAbs for 15 min at 37°C with vigorous shaking. 10 µl of freshly isolated human neutrophils were added to reach a final cell to bacteria ratio of 1:10. Phagocytosis was stopped on ice and samples were fixed with a cold 1% formaldehyde solution (10% aqueous methanol free, formaldehyde, Ultra pure; Polysciences, Inc.). Samples were acquired on a FACSVerse flow cytometer with Universal Loader (Becton Dickinson) and BD FACSuite software version 1.06. Lysis of bacteria with lysostaphin was not performed since the signal of externally bound *S. aureus* is negligible, as previously demonstrated by our group (62). In some neutrophils phagocytosis experiments bacteria are opsonized in the presence of complement source which is serum depleted of complement-fixating antibodies (denoted as ΔIgGΔIgM serum) (59) at a final concentration of 1%.

### Luminex Displacement Assay

SpAwt beads were prepared as already described above. Commercial Human IgGs (Sigma-Aldrich) were biotinylated and preincubated 30 minutes at room temperature in a 96-well Filter Plates (Millipore)at a final concentration of 8 μg/mL with vigorous shaking, to saturate 20 µl of SpAwt-coated beads. Beads were washed from excessive Hu-IgGs and then incubated with 50 μl of IgG3 mAbs 1-4 or IgG3 isotype control, serially diluted in PBS. After a final washing in PBS, the displacement of human antibodies was revealed with 50 µl of 0.1 mg/mL PE-conjugated streptavidin (Invitrogen), and was acquired with FLEXMAP 3D (Luminex Corporation). Median fluorescence intensity (MFI) was subtracted from the background signal (SpA beads + secondary antibody + streptavidin-PE) and was evaluated by using Graphpad Prism 8.1.2 (GraphPad Software Inc, CA).

## Data Availability Statement

The original contributions presented in the study are included in the article/**Supplementary Material**. Further inquiries can be directed to the corresponding author.

## Ethics Statement

For anti-SpA antibodies detection: Human sera were collected during the epidemiological study on the immune response to *S. aureus* natural infections (e-track number EPI-STAPH-007 BOD US SUPP). All subjects were recruited at Vanderbilt University Medical Center (VUMC). The study was approved by the Internal Ethical Committee and subject recruitment was approved by the VUMC Institutional Review Board. For human neutrophils isolation: Blood was obtained from healthy donors after informed consent was obtained from all subjects, in accordance with the Declaration of Helsinki. Approval from the Medical Ethics Committee of the University Medical Center Utrecht was obtained (METC protocol 07-125/C approved on March 1, 2010). The patients/participants provided their written informed consent to participate in this study.

## Author Contributions

EB, ARC, WP, CG, and AGOM performed the experiments. EB and AGOM analyzed the data. EB, AGOM, and FB wrote the paper. AGOM and FB designed the research study. SHMR, KPMK, and JAGS critically read the manuscript and provided constructive comments. All authors contributed to the article and approved the submitted version.

## Funding

This work was supported by the European Union’s Horizon 2020 research: H2020-MSCA-ITN (No. 675106 coordinated by FB, GSK, Siena, Italy) and by GSK.

## Conflict of Interest

EB is participating in a post-graduate studentship program at GSK. All authors affiliated with GSK are employees of GSK, Siena. FB holds pending and issued patents on *S. aureus* vaccine formulations.

The remaining authors declare that the research was conducted in the absence of any commercial or financial relationships that could be construed as a potential conflict of interest.

## Publisher’s Note

All claims expressed in this article are solely those of the authors and do not necessarily represent those of their affiliated organizations, or those of the publisher, the editors and the reviewers. Any product that may be evaluated in this article, or claim that may be made by its manufacturer, is not guaranteed or endorsed by the publisher.

## Supplementary Material

Supplementary Figure 1 Fitting curves to discriminate positive and negative sera for SpA opsonizing antibodies by sFcγRI signal. The figure shows the fluorescence associated to opsonized beads for every serum shown in and their respective 9 dilution points (2-fold dilution steps). A five parameter logistic curve was fitted and for all positive sera the R^2^ was reported. On the Y-axis we show the fluorescence intensity of PE-labelled, soluble Fc-gamma receptor subtracted from the background signal on a log_10_ scale. On the X-axis we show the natural logarithm of the dilution. Positive sera show the expected decreasing trend corresponding to dilution of sera. In *black* the points that fulfill the dilution step, as described in the materials and methods section, and in *red* the other ones. Negative sera display an anomalous behaviour, they do not respect the dilution step, instead display a flat curve (PHC13) or an inverse trend (44HD).

Supplementary Figure 2 Patients with high anti-SpA IgG3 display normal total IgG3 titers. Comparison between typical immunoglobulin concentration ranges in normal sera (mg/mL) (Sigma-Aldrich, Reference: https://www.sigmaaldrich.com/deepweb/assets/sigmaaldrich/marketing/global/documents/326/744/normalimmunoranges.pdf. Accessed: 2021-07-02) and concentration of IgG subclasses tested in sera.

Supplementary Figure 3 Detection of subclass 1 anti-SpA opsonizing antibodies deposited on SpA wild-type coated beads. Upon opsonization of SpAwt beads, we detected human IgGs with Rat anti-Human IgG1. The data clearly show that it is not possible to discriminate positive or negative results from the curves, contrarily to what detected in and **S1**. The data represent one exemplificative experiment.

Supplementary Figure 4 THP-1 phagocytosis of SpAwt beads mediated by anti-SpA model mAbs. Single mAbs comparison.

Supplementary Figure 5 THP-1 phagocytosis of GFP USA300 mediated by anti-SpA model mAbs. Single mAbs comparison.

Supplementary Figure 6 Binding assay of anti-SpA model mAbs to USA300 lac wt. Grouped and single mAbs view.

Supplementary Figure 7 Neutrophil phagocytosis of GFP USA300 mediated by anti-SpA model mAbs Single mAbs comparison.

Supplementary Figure 8 THP-1 phagocytosis of GFP USA300 pre-incubated with human IgGs, mediated by anti-SpA model mAbs. Single mAbs comparison.

Supplementary Figure 9 SPR sensograms showing the binding kinetics of each anti-SpA mAb to SpA_KK_ and SpA_AA_. Surface plasmon resonance sensograms showing the binding kinetics within a 200s frame of the four IgG3 model anti-SpA antibodies to either 5-domain SpA_KK,_ in which Fc-binding properties are abolished (*green*) or 5-domain SpA_AA_, in which VH3-binding properties are abolished (*red*).

Supplementary Figure 10 Amino acid sequences of model anti-SpA mAbs used. mAb-1 and isotype controls were produced starting from CDR information reported in (21), mAbs 2-4 were produced starting from CDR information reported in (54).

Supplementary Figure 11 Amino acid sequence of SpAwt and SpAmut. Amino acid sequence of SpAwt expressed by *S. aureus* strain NCTC 8325, Locus_tag SAOUHSC_00069, GI (GenInfo Identifier assigned by NCBI): 88193885. Here is reported the original sequence of the protein, as well as the construct for its expression in *E. coli*. The colors indicate: the secretion signal (green), the 5 IgG binding regions (yellow), the region X repeats (red), anchor for sortase (white), the LPXTG motif for the sortase enzyme (blue). The amino acid sequence of SpAmut was generated as indicated in indicated in the following patent (66).

## Abbreviations

C1, Complement protein 1; CDR, Complementarity determining regions; CR, Complement receptor; Fab, Fragment, antigen-binding; Fc, Fragment, crystallizable; FcγR, Fc gamma receptor; IgG, Immunoglobulin G; mAb, Monoclonal antibody; MRSA, Methicillin-resistant *S. aureus*; OR, Odd ratio; SpA, Staphylococcal Protein A; TNFR, Tumor Necrosis Factor α Receptor; VRSA, Vancomycin-resistant *S. aureus*; vWF, *von Willebrand* factor.
