# Supplementary figures and images for: Natural Human Immunity Against Staphylococcal Protein A Relies on Effector Functions Triggered by IgG3
Source: Front Immunol. 2022 Mar 11;13:834711. doi: 10.3389/fimmu.2022.834711 (PMC8963248; doi:10.3389/fimmu.2022.834711)

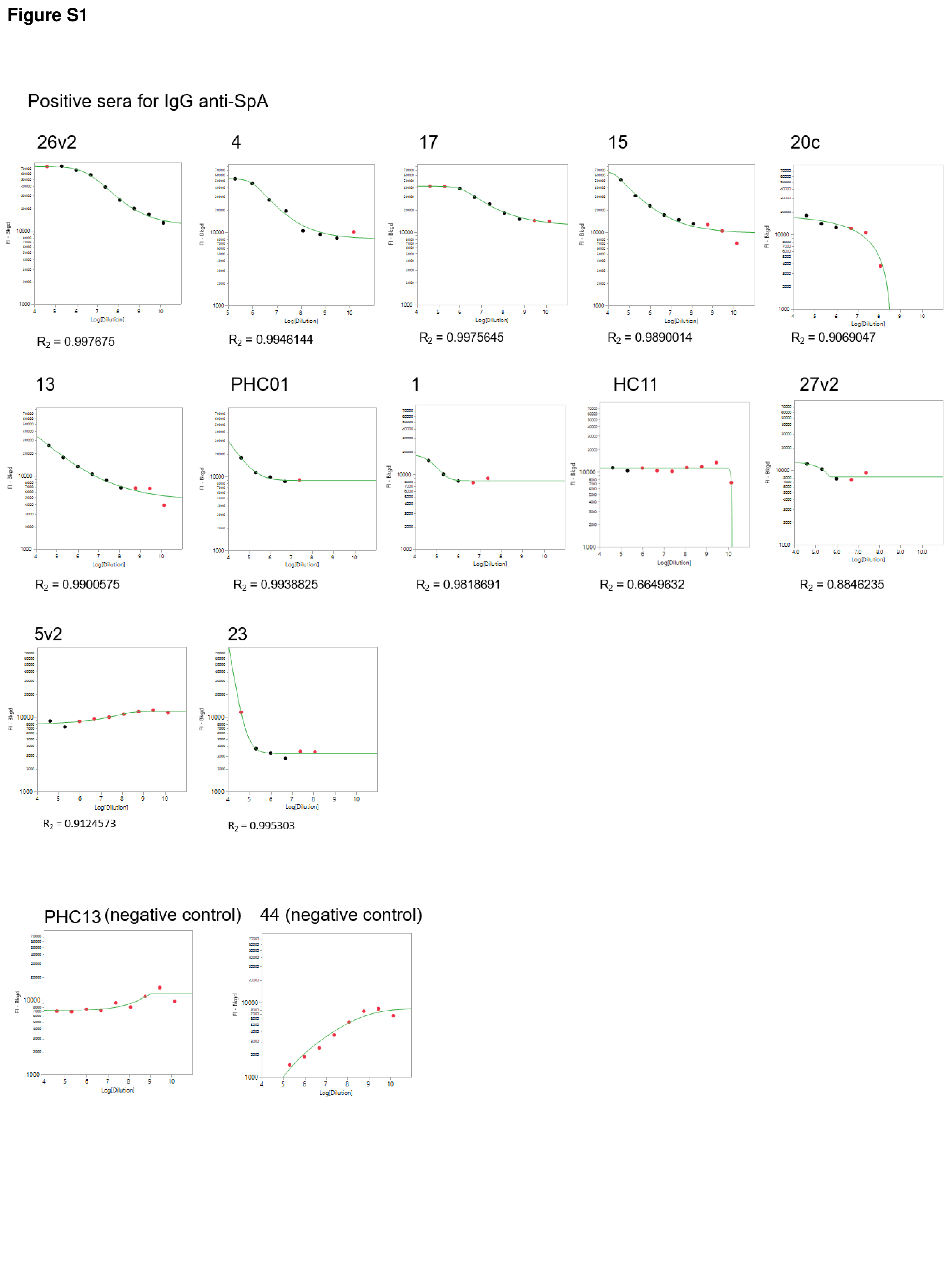

Supplement: Supplementary Figure 1 — Fitting curves to discriminate positive and negative sera for SpA opsonizing antibodies by sFcγRI signal. The figure shows the fluorescence associated to opsonized beads for every serum shown in and their respective 9 dilution points (2-fold dilution steps). A five parameter logistic curve was fitted and for all positive sera the R2 was reported. On the Y-axis we show the fluorescence intensity of PE-labelled, soluble Fc-gamma receptor subtracted from the background signal on a log10 scale. On the X-axis we show the natural logarithm of the dilution. Positive sera show the expected decreasing trend corresponding to dilution of sera. In black the points that fulfill the dilution step, as described in the materials and methods section, and in red the other ones. Negative sera display an anomalous behaviour, they do not respect the dilution step, instead display a flat curve (PHC13) or an inverse trend (44HD). [file Image_1.jpeg]

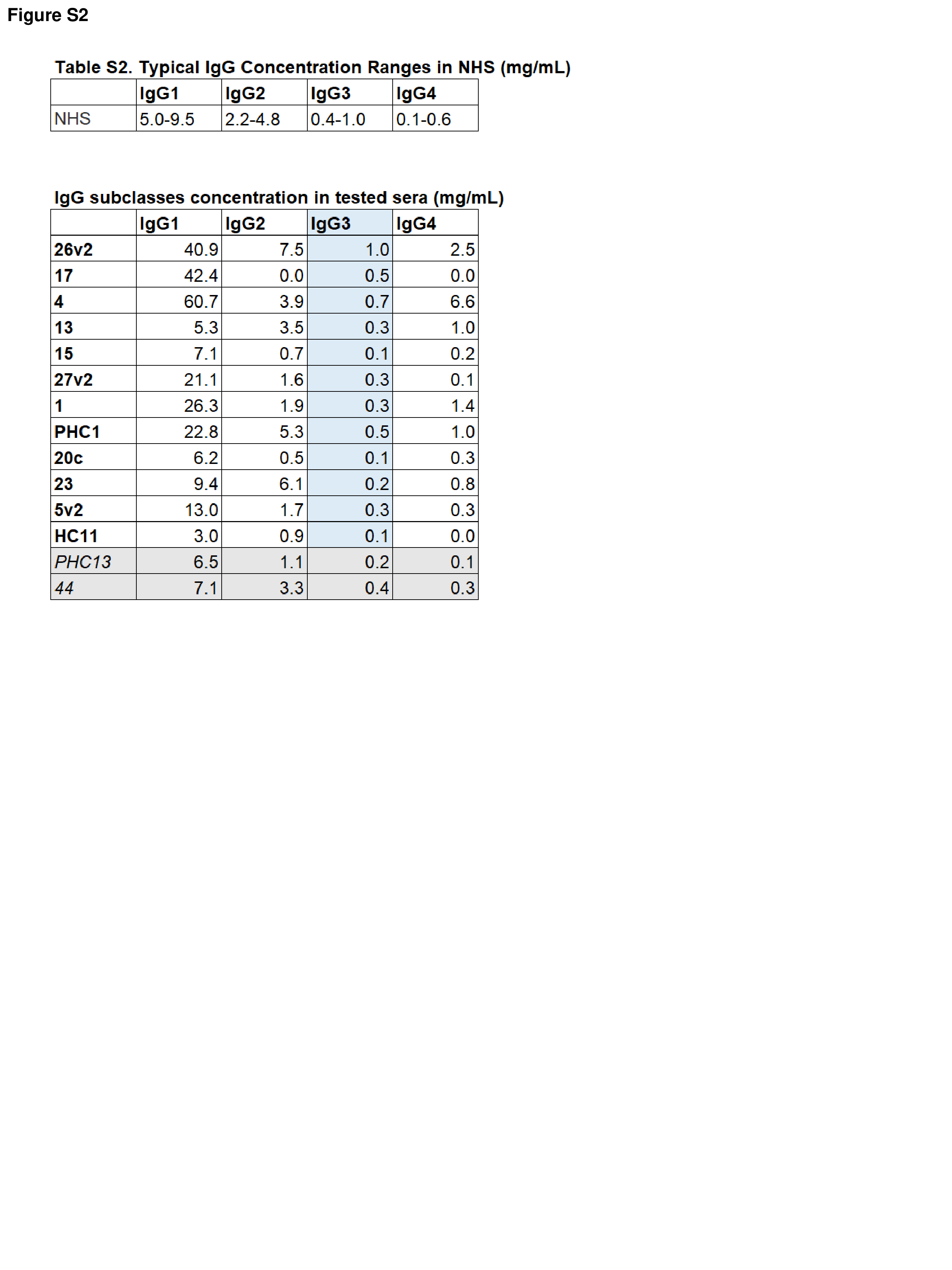

Supplement: Supplementary Figure 2 — Patients with high anti-SpA IgG3 display normal total IgG3 titers. Comparison between typical immunoglobulin concentration ranges in normal sera (mg/mL) (Sigma-Aldrich, Reference: https://www.sigmaaldrich.com/deepweb/assets/sigmaaldrich/marketing/global/documents/326/744/normalimmunoranges.pdf. Accessed: 2021-07-02) and concentration of IgG subclasses tested in sera. [file Image_2.jpeg]

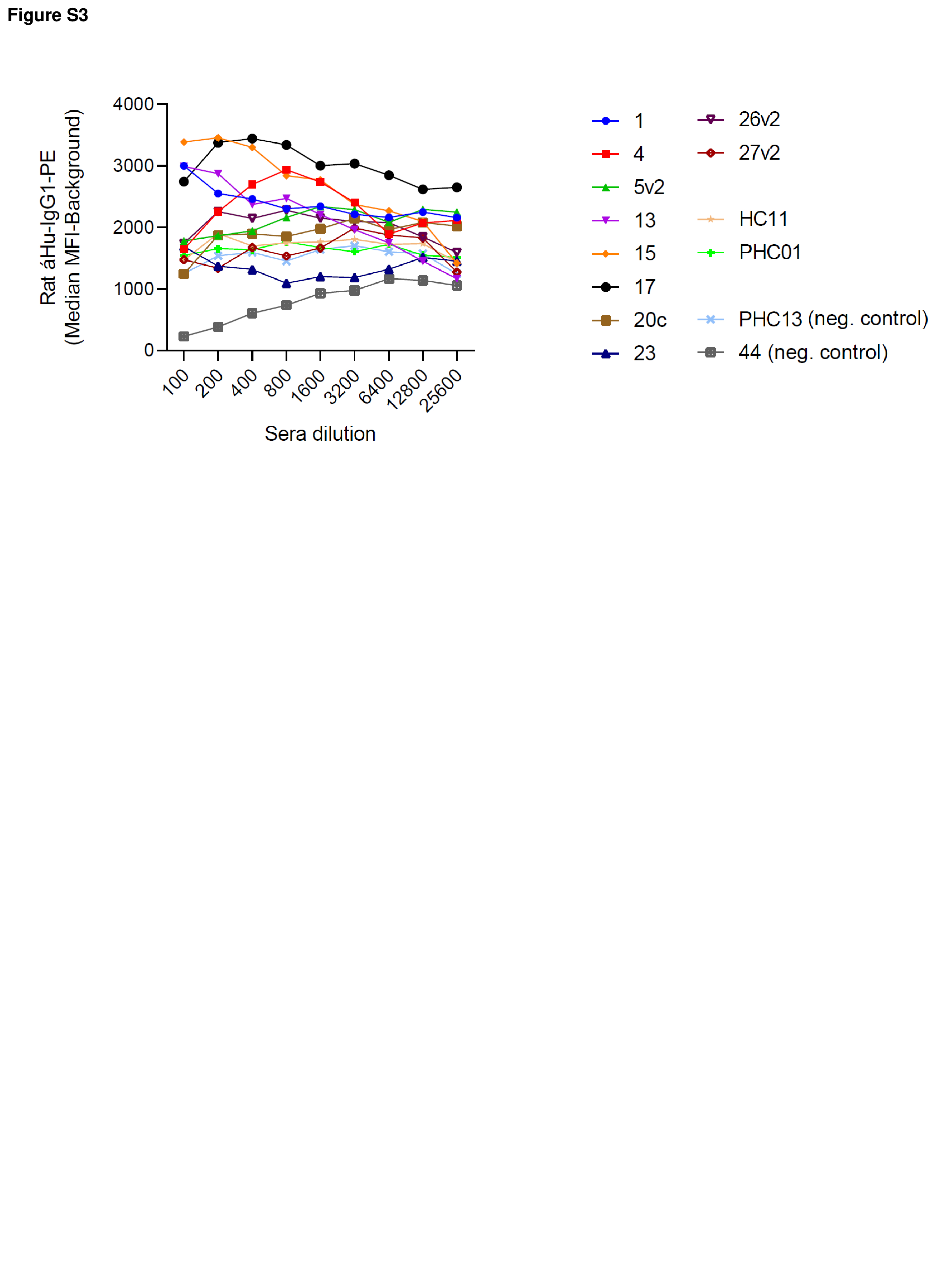

Supplement: Supplementary Figure 3 — Detection of subclass 1 anti-SpA opsonizing antibodies deposited on SpA wild-type coated beads. Upon opsonization of SpAwt beads, we detected human IgGs with Rat anti-Human IgG1. The data clearly show that it is not possible to discriminate positive or negative results from the curves, contrarily to what detected in and S1 . The data represent one exemplificative experiment. [file Image_3.jpeg]

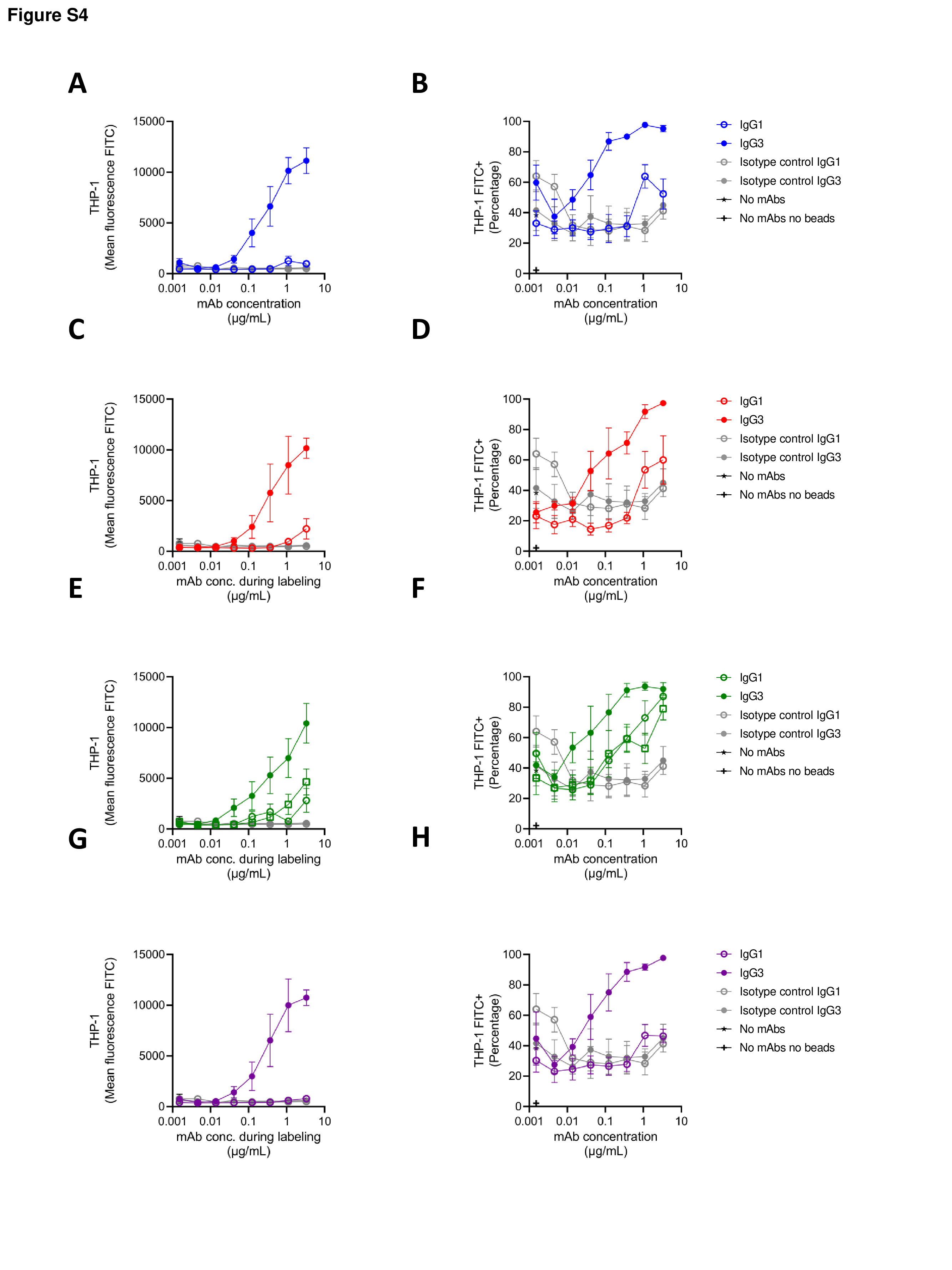

Supplement: Supplementary Figure 4 — THP-1 phagocytosis of SpAwt beads mediated by anti-SpA model mAbs. Single mAbs comparison. [file Image_4.jpeg]

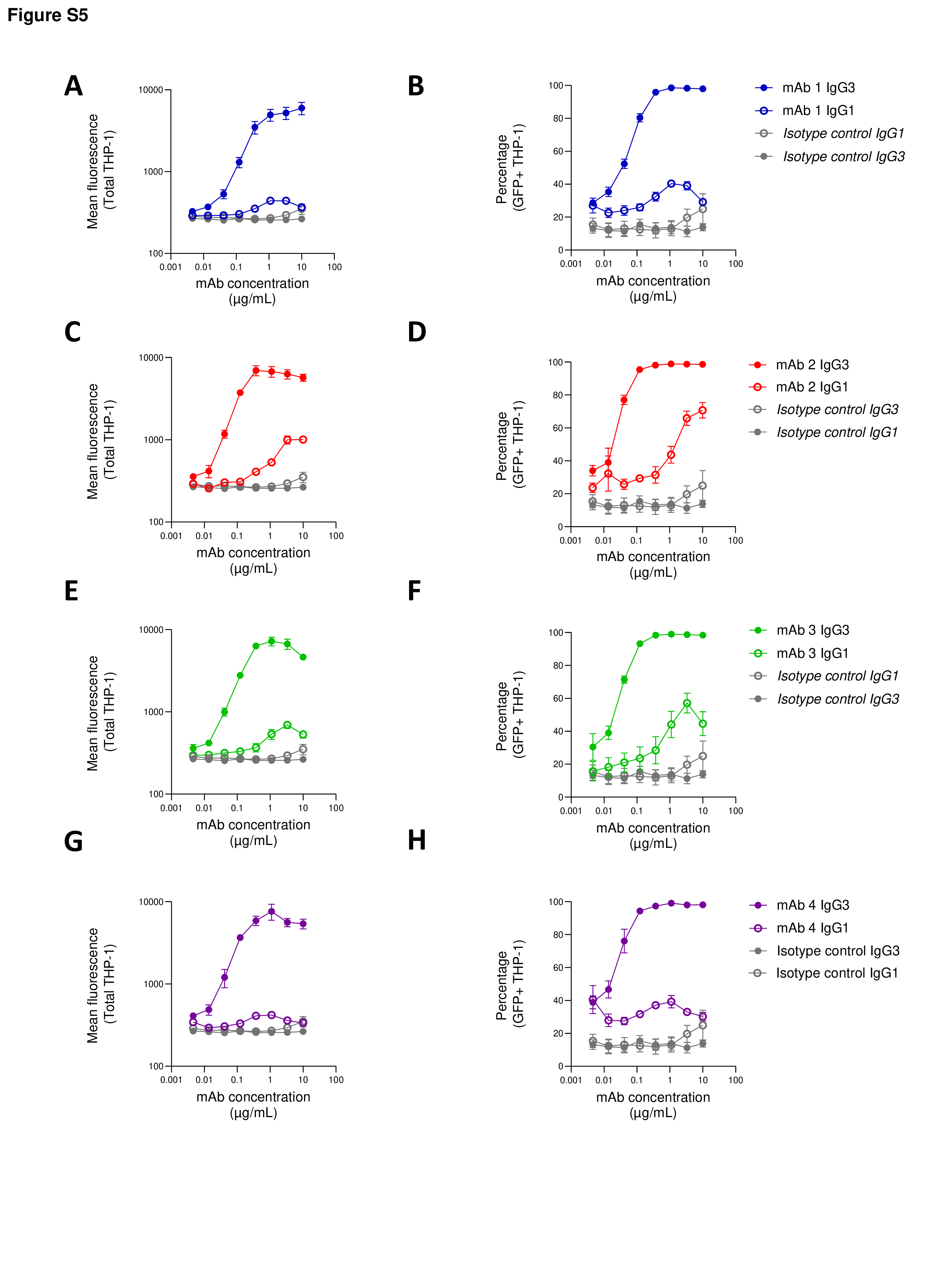

Supplement: Supplementary Figure 5 — THP-1 phagocytosis of GFP USA300 mediated by anti-SpA model mAbs. Single mAbs comparison. [file Image_5.jpeg]

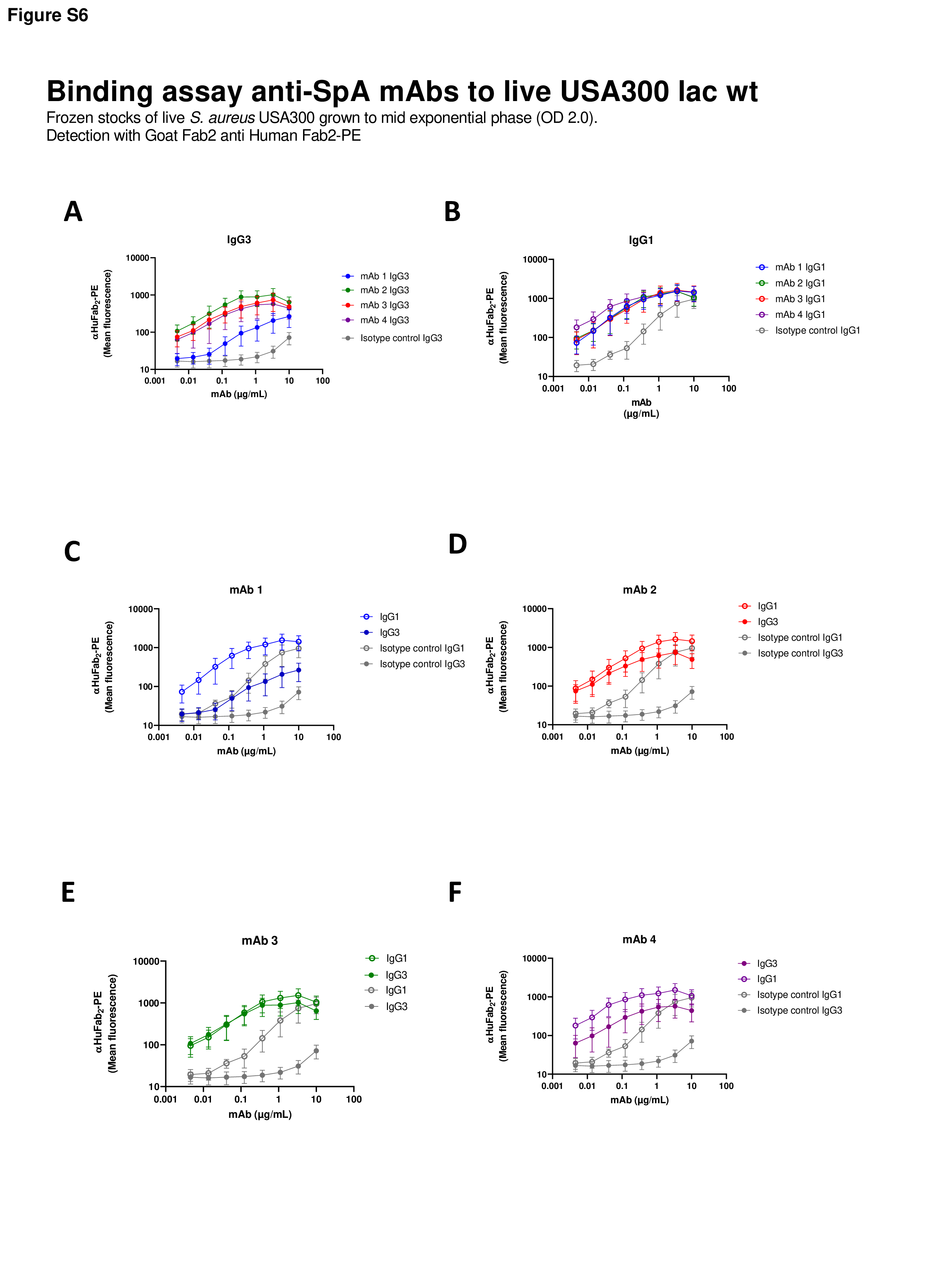

Supplement: Supplementary Figure 6 — Binding assay of anti-SpA model mAbs to USA300 lac wt. Grouped and single mAbs view. [file Image_6.jpeg]

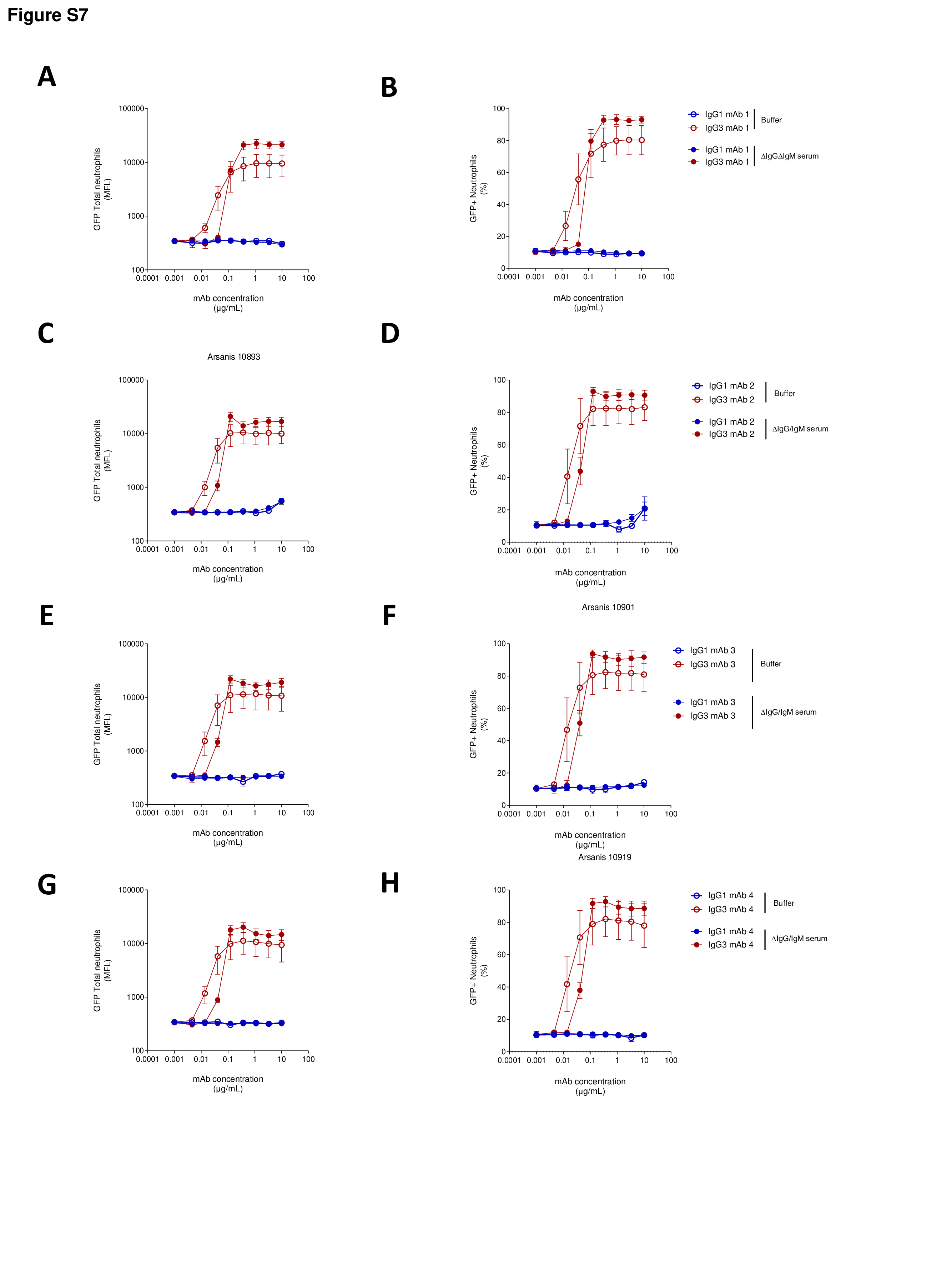

Supplement: Supplementary Figure 7 — Neutrophil phagocytosis of GFP USA300 mediated by anti-SpA model mAbs Single mAbs comparison. [file Image_7.jpeg]

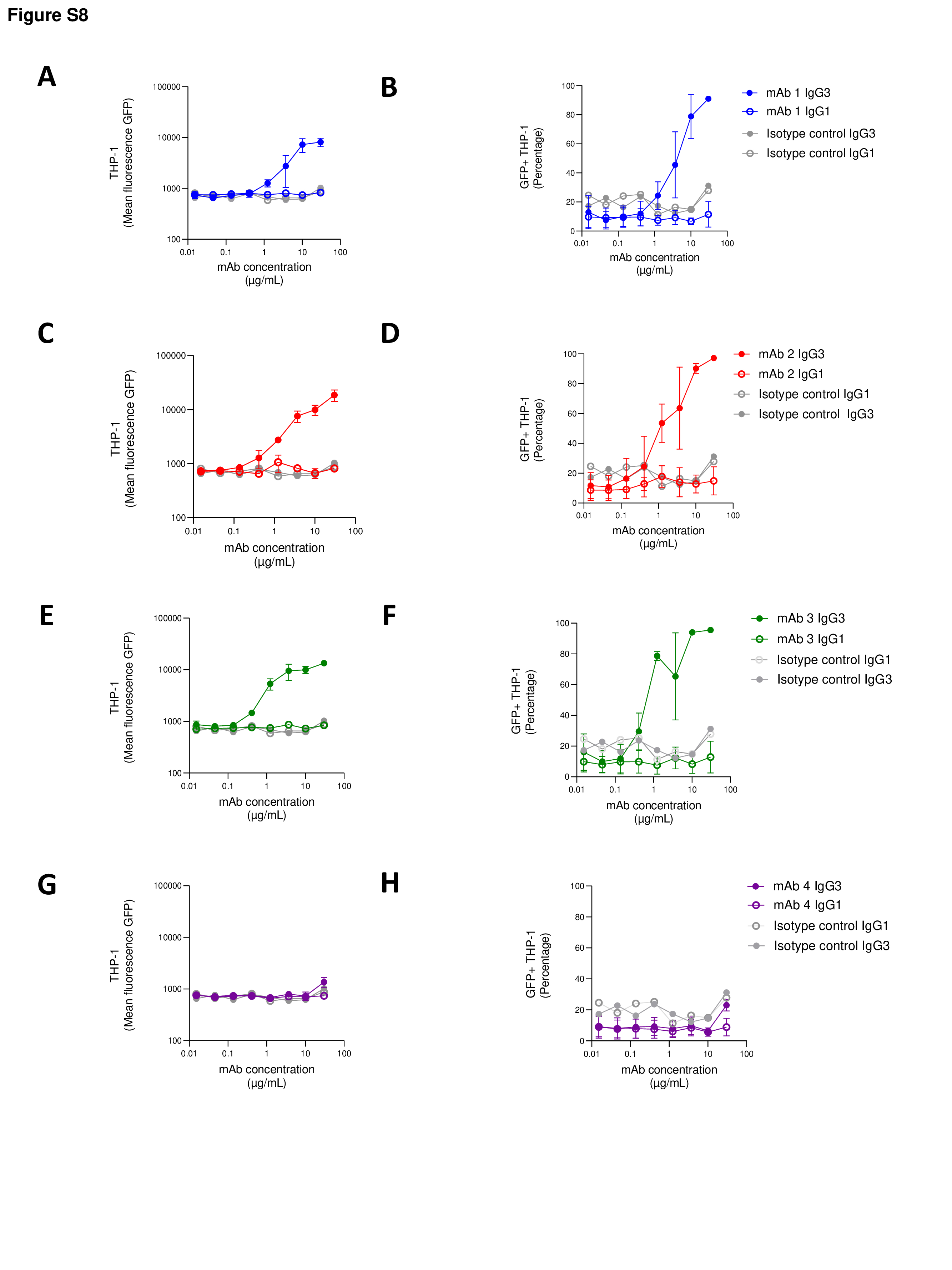

Supplement: Supplementary Figure 8 — THP-1 phagocytosis of GFP USA300 pre-incubated with human IgGs, mediated by anti-SpA model mAbs. Single mAbs comparison. [file Image_8.jpeg]

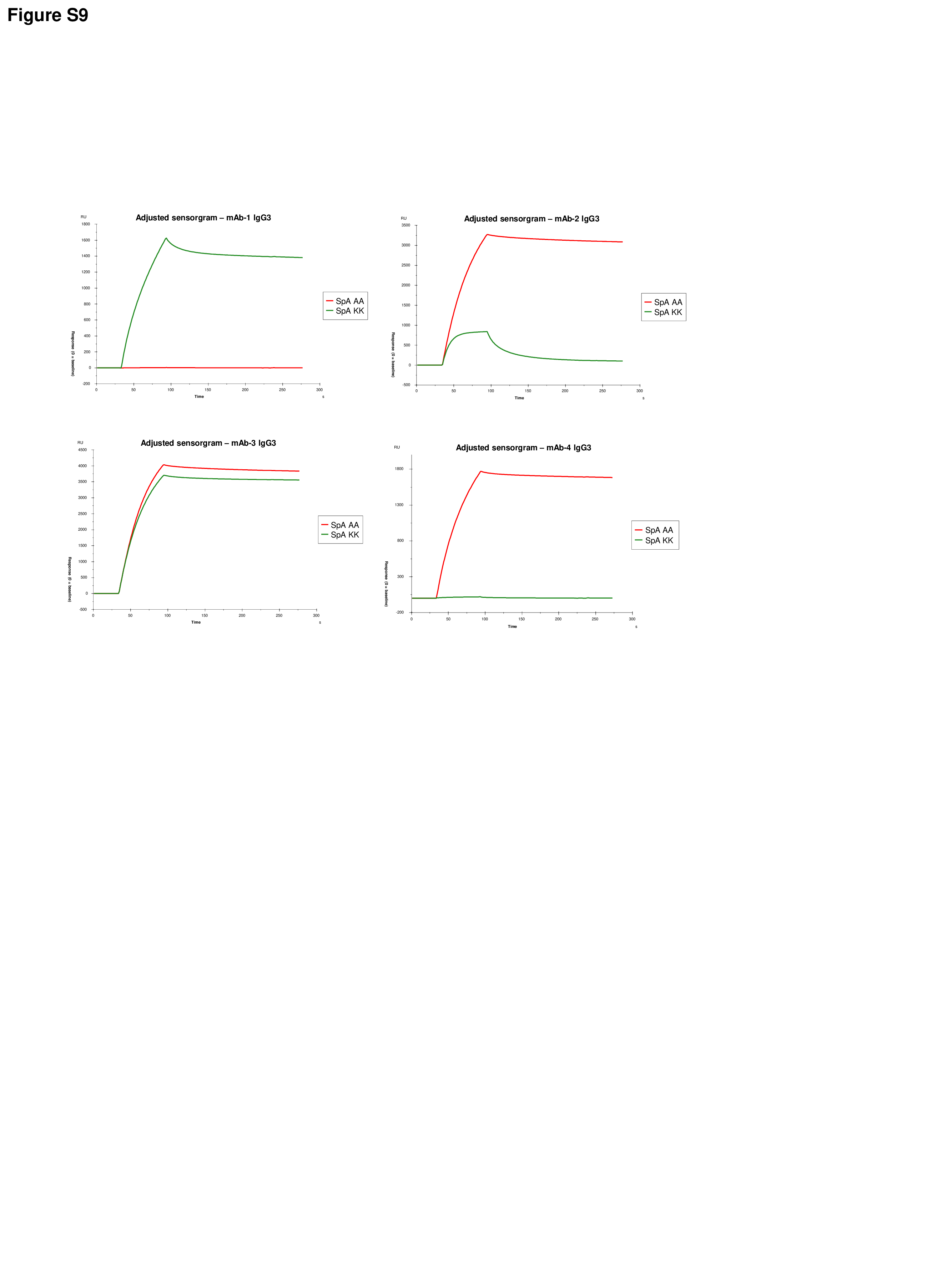

Supplement: Supplementary Figure 9 — SPR sensograms showing the binding kinetics of each anti-SpA mAb to SpAKK and SpAAA. Surface plasmon resonance sensograms showing the binding kinetics within a 200s frame of the four IgG3 model anti-SpA antibodies to either 5-domain SpAKK, in which Fc-binding properties are abolished (green) or 5-domain SpAAA, in which VH3-binding properties are abolished (red). [file Image_9.jpeg]

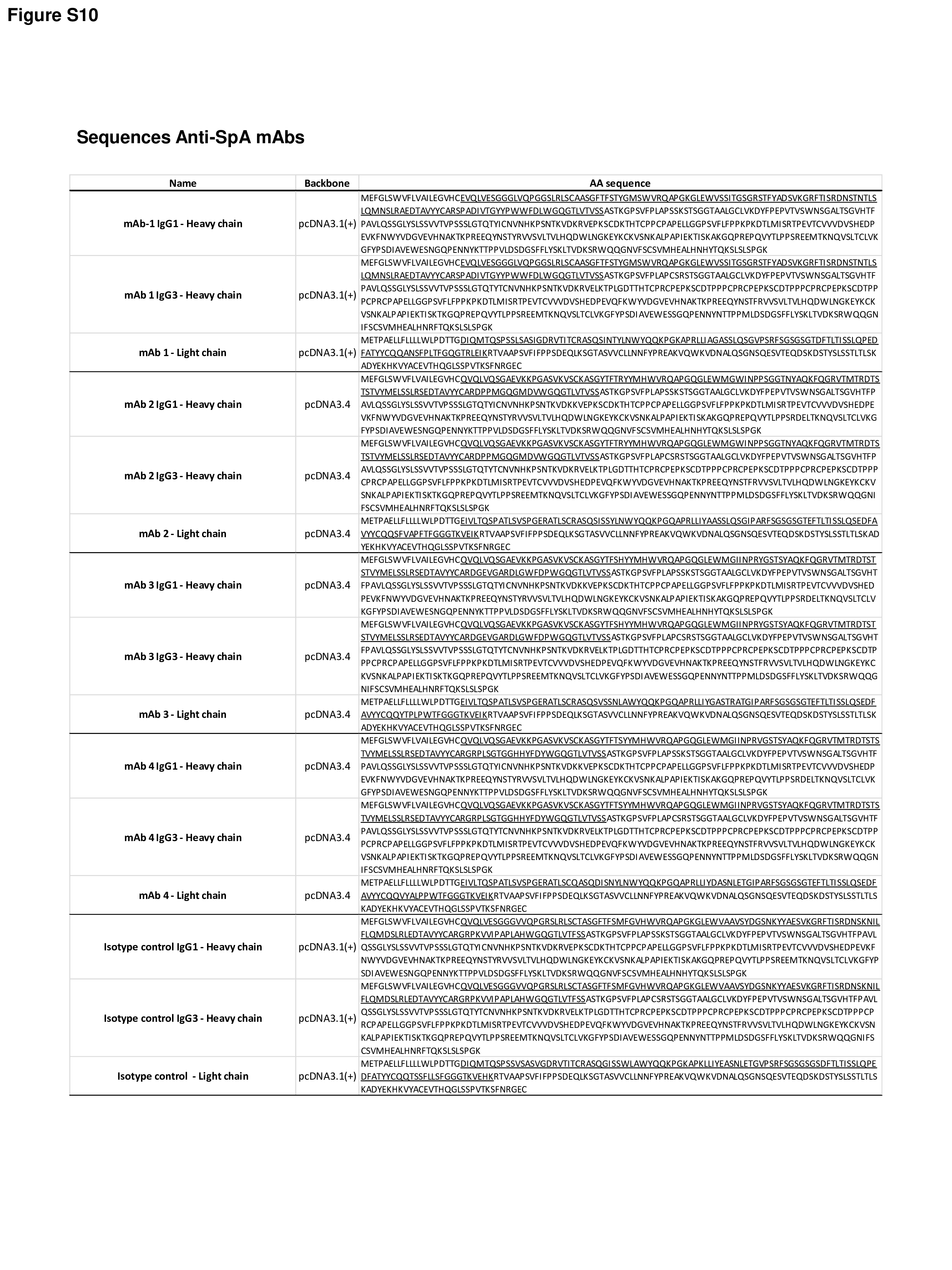

Supplement: Supplementary Figure 10 — Amino acid sequences of model anti-SpA mAbs used. mAb-1 and isotype controls were produced starting from CDR information reported in (21), mAbs 2-4 were produced starting from CDR information reported in (54). [file Image_10.jpeg]

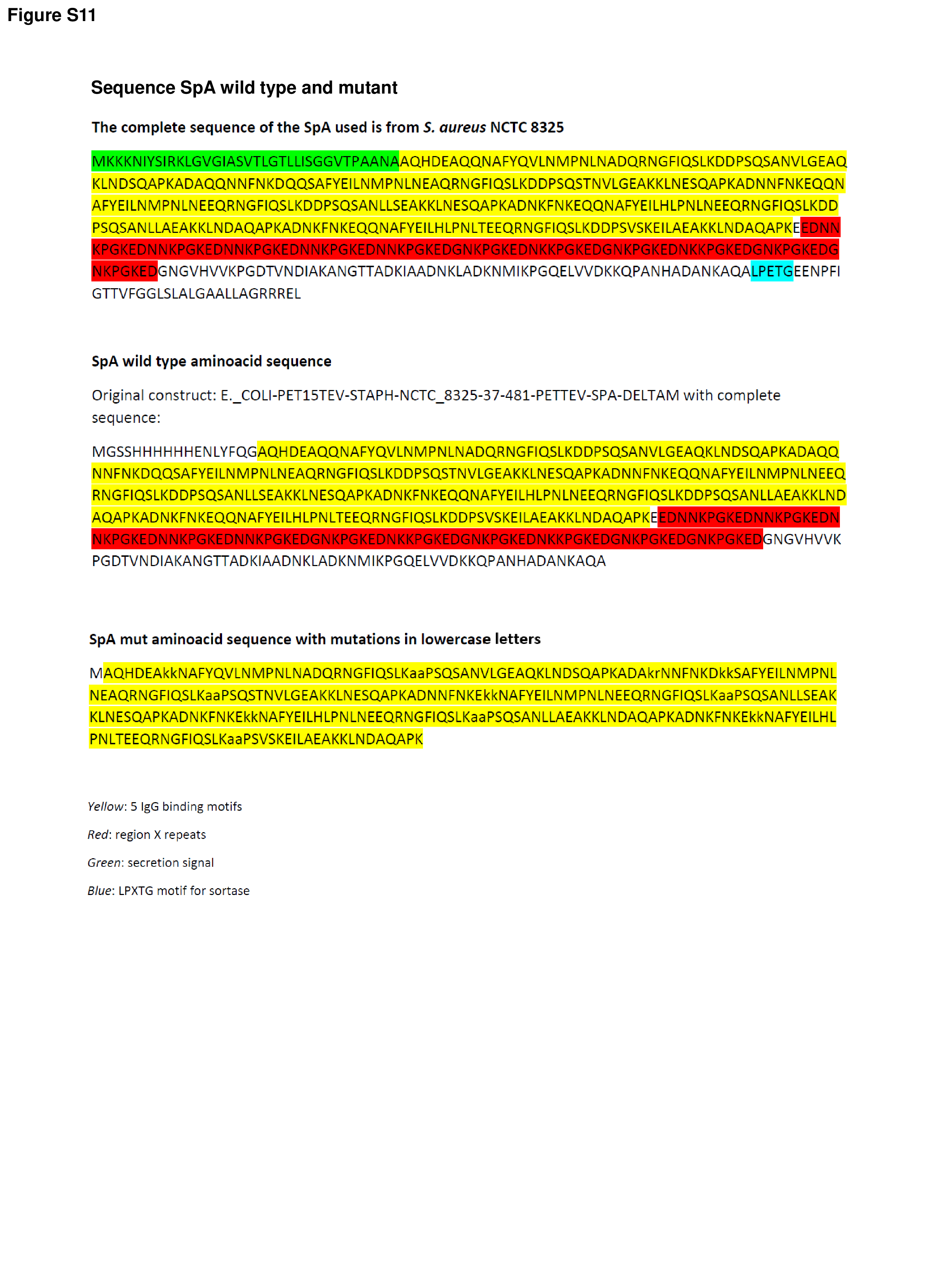

Supplement: Supplementary Figure 11 — Amino acid sequence of SpAwt and SpAmut. Amino acid sequence of SpAwt expressed by S. aureus strain NCTC 8325, Locus_tag SAOUHSC_00069, GI (GenInfo Identifier assigned by NCBI): 88193885. Here is reported the original sequence of the protein, as well as the construct for its expression in E. coli. The colors indicate: the secretion signal (green), the 5 IgG binding regions (yellow), the region X repeats (red), anchor for sortase (white), the LPXTG motif for the sortase enzyme (blue). The amino acid sequence of SpAmut was generated as indicated in indicated in the following patent (66). [file Image_11.jpeg]
